# Nutrition Report Cards: An Opportunity to Improve School Lunch Selection

**DOI:** 10.1371/journal.pone.0072008

**Published:** 2013-10-02

**Authors:** Brian Wansink, David R. Just, Richard W. Patterson, Laura E. Smith

**Affiliations:** 1 John S. Dyson Professor of Marketing at the Dyson School of Applied Economics and Management, Cornell University, Ithaca, New York, United States of America; 2 Associate Professor, Dyson School of Applied Economics and Management at Cornell University, Ithaca, New York, United States of America; 3 Doctoral Candidate in Policy Analysis and Management, Cornell University, Ithaca, New York, United States of America; 4 Doctoral Candidate in Nutritional Sciences, Cornell University, Ithaca, New York, United States of America; INRA, France

## Abstract

**Objective:**

To explore the feasibility and implementation efficiency of Nutritional Report Cards(NRCs) in helping children make healthier food choices at school.

**Methods:**

Pilot testing was conducted in a rural New York school district (K-12). Over a five-week period, 27 parents received a weekly e-mail containing a NRC listing how many meal components (fruits, vegetables, starches, milk), snacks, and a-la-carte foods their child selected. We analyzed choices of students in the NRC group vs. the control group, both prior to and during the intervention period. Point-of-sale system data for a-la-carte items was analyzed using Generalized Least Squares regressions with clustered standard errors.

**Results:**

NRCs encouraged more home conversations about nutrition and more awareness of food selections. Despite the small sample, the NRC was associated with reduced selection of some items, such as the percentage of those selecting cookies which decreased from 14.3 to 6.5 percent. Additionally, despite requiring new keys on the check-out registers to generate the NRC, checkout times increased by only 0.16 seconds per transaction, and compiling and sending the NRCs required a total weekly investment of 30 minutes of staff time.

**Conclusions:**

This test of concept suggests that NRCs are a feasible and inexpensive tool to guide children towards healthier choices.

## Introduction

A healthy diet is a key component of child health and development. Yet, many American children have poor diets, consuming too many calories [1], [2], too few vitamins and minerals [3], and too few fruits and vegetables [4]. With more than 70% of children in grades K-12 eating at least three lunches per week provided by the National School Lunch Program (NSLP) [3], the content of those lunches is a logical target for improving child nutrition. While directly targeting students can lead to better lunchtime choices, studies suggest that involving students' parents can enhance the positive outcomes of such nutrition interventions [5].

In this article, we evaluate whether the “Nutrition Report Card” may be a low-cost, scalable intervention providing parents with information about which foods their child orders at school. Despite its name, the Nutrition Report Card (NRC) does not provide a grade or evaluation of food choices, but rather, simply records which foods are selected over a given time period. For example, it could list how many fruits, vegetables, starches, and snacks were selected and if white or flavored milk was chosen. Even without any action on the part of parents, the NRC could positively influence food choice through the child's perception that parents were observing those choices [6]. At the other side of the spectrum, more engaged parents might use the NRC to set eating goals with their child or to limit their child's a-la-carte purchases [7]. In the mid-range of possible parental responses, the NRC could prompt questions and information-sharing between parents and children on the topics of school lunches and food choice [8], [9]. In a recent study, Kaplan, Kernan, and James [10], found that many parents and grandparents would like more opportunities to discuss nutrition behavior with children. The NRC could provide a catalyst for such discussions.

The NRC is based upon electronic purchasing records and could be e-mailed regularly to parents at little cost to the school. Research suggests that even passive feedback and follow-up increases success of weight loss and weight maintenance in adults [11], [12]. Analogously, Nutrition Report Cards sent weekly or monthly could perhaps improve the success and maintenance of nutrition goals for children by providing regular feedback and improving communication between school and home.

The NRC is similar in concept to the Body Mass Index (BMI) Report Card that Arkansas schools initiated. Since 2003, the Arkansas schools have clinically measured and weighed students annually and sent a BMI Report Card to parents [13], [14], [15], [16]. While the impact on weight loss is not clear [13], [16], research suggest that BMI Report Cards may encourage increases in child exercise [14] and help parents more accurately assess their child's health [15]. BMI Report Cards remain controversial, however, with disagreements on whether they increase social stigma and negative eating behaviors, such as skipping meals, binge eating, or purging [13], [14]. Schools interested in implementing the Nutrition Report Card should be cautious of the risks associated with sending sensitive information to parents and should create NRC's that are easy to understand, avoid stigmatizing language, and contain actionable information.

## Methods

### Enrolling participants in the study

After obtaining Cornell University Institutional Review Board approval, a letter was mailed to all parents in a rural school district in New York inviting them to enroll their child in the study. Physical letters were used because the school district did not maintain an e-mail list for parents. The letter explained that the study would examine child nutrition, but did not specify the nature of the intervention until they enrolled their child. Interested parents were directed to a website to enter their e-mail address, their child's information, and consent to the specifics of the study. Due to limited time available for the study, no reminder or follow-up contacts were made and no incentives were offered for participation. This led to a relatively small number of participants. Our sample includes students (n = 35) ranging from grades K-12.

### Obtaining Data from Schools

Most schools in the United States use computerized point of sale (POS) systems to record student purchases and maintain account balances. While POS systems record purchases with varying specificity, almost all systems are capable of outputting per-item data with student, date, and cost identified. Data recorded in this format would enable schools to generate NRCs similar to those created for this study. While some POS systems are already configured to record meal components, most indicate only if a reimbursable NSLP lunch was purchased. An important step in implementing NRCs is learning which lunch components a district already records and which one the district has the capacity to record.

In the study school district, computer systems were configured to record reimbursable meals and the specific names of a-la-carte items prior to our intervention. For the purposes of this study, cash register keyboards were reconfigured by adding three virtual buttons to track selection of fruit/vegetable items, starchy sides, and white milk. These three components were recorded whether purchased as a la carte items, or as part of a reimbursable meal. However, these three buttons were added at the same time as the NRC was implemented. Thus, difference-in-difference style results could only be calculated for a-la-carte items. Cashiers were trained to identify fruit/vegetables and starchy sides (e.g. tater-tots and French fries) within each meal and to press the corresponding virtual buttons. Programming additional transaction keys into POS software systems is a simple task, generally taking less than one hour. In some cases, however, it may be complicated, time-consuming, or even impossible. Even in cases where reconfiguration is simple, adding keystrokes for purposes of data collection increases the time it takes cashiers to conduct each transaction. Thus, to avoid lengthening the lunch payment process, there should be a limited number of food categories that cashiers are asked to identify and record. For this reason, we recorded fruit and vegetable selection in a single category. Additionally, we measured transaction times before and after keys were added to assess the impact of the NRC intervention on transaction times.

### Turning POS data into Nutrition Report Cards

For the five weeks of the study, purchase data for all students were recorded for analysis. Purchase data for students enrolled in the study were compiled in a Nutrition Report Card sent weekly to their parents by e-mail. The NRC did not grade the student's food choices in any way, but simply noted what had been purchased. For each day of the week the NRC listed 1) whether the student had purchased an NSLP meal, 2) whether the meal included fruits/vegetables, starchy sides, white milk, flavored milk, and 3) each a-la-carte item. The school district's POS system was programmed to record a-la-carte items by name (e.g., cookies, chips or ice cream) due to the item-specific variation in price. Thus, the NRC could provide a precise list of a-la-carte items purchased, whereas items included in the NSLP could only be roughly categorized. The NRC did not indicate if the student had purchased food from vending machines or other food outlets within the school grounds but beyond the lunchroom.

### Data and Methods for Analysis

Data for this study include grade-level, transaction time for each student purchase, a-la-carte purchases, and meal components selected for each student in treatment (n = 35) and control (n = 1,460). The grade distribution of students (grades K-12) was similar between treatment (mean = 5.63, sd = 3.40) and control (mean = 5.65, sd = 3.68). Data for each transaction included a time stamp that identified the day, hour, minute, and second of the purchase. Transaction times were computed by measuring the time, in seconds, between purchases within a school. To avoid mistaking infrequent purchases for slow transaction times, we focus on transactions taking fewer than 20 seconds (84% of both pre-post POS key-change transactions). A-la-carte purchases of cookies, ice cream, and chips were recorded both before and after key changes and NRC treatments and are summarized in Table 1. Because the changes to POS systems and creation of NRCs were implemented simultaneously, student purchase data on fruit/vegetables, starchy sides and milk were not available prior to the study period.

**Table 1:** Purchase Incidence of Selected Lunch Foods.

	Control Group		Treatment Group	
	Pre-NRC	Post-NRC	Pre-NRC	Post-NRC
	n = 1417*	n = 1398*	n = 35*	n = 35*
Variables	t = 32295	t = 25803	t = 789	t = 603
Grade(K-12)	5.409	5.340	5.260	5.313
	(3.534)	(3.498)	(3.261)	(3.216)
Cookies	0.115	0.107	0.143	0.065
	(0.416)	(0.421)	(0.429)	(0.272)
Ice Cream	0.090	0.095	0.076	0.090
	(0.290)	(0.298)	(0.270)	(0.297)
Chips	0.103	0.079	0.089	0.038
	(0.352)	(0.309)	(0.296)	(0.210)
Fruit	.	1.009	.	1.098
	.	(0.720)	.	(0.808)
Starch	.	0.115	.	0.129
	.	(0.321)	.	(0.346)
White Milk	.	0.091	.	0.116
	.	(0.288)	.	(0.321)
Flavored Milk	.	0.857	.	0.849
	.	0.353	.	0.358

SD in parentheses, n = number of participants, t = number of transactions. *Chips only sold in grades 7-12. For chips--Control Pre-NRC n = 590/t = 12719, Control Post-NRC n = 578/t = 9970, Treatment Pre-NRC n = 15/t = 316, and Treatment Post-NRC n = 15/t = 265.

To analyze the impact of changing POS systems on transaction times, we compare transaction times in the weeks preceding and following the system change. Analysis of the impact of the NRC on children's food selection, however, is less clear. One should note that parents who chose to enroll their children in the NRC may be different from those who did not enroll their children. Reports of differences in purchasing behavior based upon the NRC are only suggestive and cannot be interpreted as in a true controlled experiment. Our preliminary analysis of the relationship between enrollment in NRCs and a-la-carte selection was examined by using difference-in-difference ordinary least squares (OLS) regressions with standard errors clustered by student.

## Results

The first objective of the Nutrition Report Card was to investigate whether providing information to parents about the components of their child's school lunch holds any promise of improving what students select for their lunch. A second objective was to investigate the implementation feasibility of the NRC and its impact on transaction times.

Despite the small sample size, Table 1 suggests that providing parents with a NRC may impact what lunch foods their child selects. For example, those in the NRC treatment group had a purchase incidence of 0.143 cookies per day prior to the intervention and 0.065 afterwards (a difference-in-difference of 0.07; p = 0.03). Students in the treatment group also selected fruits and vegetables more frequently and flavored milk less frequently. Neither of these differences, however, was statistically significant. A full analysis taking pre-treatment behavior into account can only be conducted on a la carte items (Table 2). Changes in ice cream and chip purchases were not significantly affected by the NRC treatment.

**Table 2:** Nutrition Report Card Reduces Cookie Selection, but Does Not Significantly Change Ice Cream or Chip Selection.

	Difference-in- difference estimates of effect of NRC on selection		
	Cookies	Ice Cream	Chips†
	n = 1495	n = 1495	n = 633
Variable	t = 59490	t = 59490	t = 23270
Treated*Post Treatment	−0.070**	0.008	−0.026
	(0.033)	(0.013)	(0.035)
Treated Group	0.030	−0.014	−0.017
	(0.036)	(0.017)	(0.041)
Post Treatment	−0.008	0.005*	−0.024***
	(0.005)	(0.003)	(0.005)
Constant	0.113	0.090	0.106
	(0.007)	(0.004)	(0.012)

OLS Coefficients reported, robust standard errors clustered by individual. †Chips only available to students in grades 7–12.

*** p<0.01.

** p<0.05.

* p<0.10.

A post-intervention survey (n = 22 of 35 surveyed) indicated that the NRC encouraged some parents to have nutrition conversations with their children and that they increased children's awareness that their lunch selections were being observed. In open-ended responses, parents expressed appreciation for knowing what their children were eating and reported that the NRCs altered what they served at family meals. Some parents used this as an opportunity for nutrition education (e.g. “Keeping track of what my children were purchasing at school was helpful in talking with them about making better choices about food”). Other parents were interested for economic reasons (e.g. “I liked seeing the snacks they purchased. It made me understand why my one son was always out of money on his account”).

A second objective was to investigate the impact of the NRC on transaction times. In the first week, transaction times increase from 6.93 seconds to 7.63 seconds. However, the difference in transaction times decreased steadily over the six-week period to the point where transactions took only 0.16 seconds longer in the fifth week after the change. These findings suggest that adding keys to generate detailed NRCs added only 16 seconds of staff time per 100 students, after five weeks. Additionally, a total of about 30 minutes of staff time each week was required to process data, generate report cards, check quality, and send to parents.

### Discussion and Implications for Student Health

This pilot study underscores that a NRC intervention is feasible and efficient. Additionally, while this study was not designed to test the effectiveness of NRC, preliminary results provide hope for their capacity to improve children's food selection. Although the results are preliminary, they suggest that NRCs may be helpful in nudging children towards more healthy, less expensive options and away from less healthy, more expensive ones and to do so at little cost to the school district. Future research should use random assignment and full pre-treatment and post-treatment data collection to rigorously test the effectiveness of NRCs in various types of schools (rural, suburban, and inner city) and at various grade levels (elementary, middle, and high school).

The results of our study related to effectiveness must be cautiously interpreted. Because parents selected their child into the treatment condition, differences in behavior between treatment and control groups may be due to differences in the types of families that would opt into a NRC and those that would not. Thus, our results may overstate the impact of the NRC. This is less of a concern for a-la-carte items for which we have baseline consumption data. This baseline allows us to use a difference-in-differences approach, which can mitigate some of the potential selection bias. Nonetheless, a true test of effectiveness should involve random assignment to treatment. Finally, it should be noted that some families-particularly those of low socio-economic status-may not have access to e-mail at home. This would limit the effectiveness of NRCs to reach some of the primary cohorts of at-risk children.

If proven effective in a larger-scale randomized trial, the NRC could be easy and cost-effective for schools to implement. For schools with POS systems ready to output student lunch data and communicate with parents via e-mail, implementing a NRC would require minimal programming and ongoing management. Another attractive component of a NRC is the scalability of such an intervention. Because data processing, report card generation, and email sending are each automated, increasing the number of NRCs a school sends would require little, if any, additional time for NRC administrators. A district providing NRCs for some of its schools could easily add other schools, provided that their data could be formatted similarly. Along with ease of implementation and scalability, NRCs have the attractive feature of engaging parents in their child's decision-making process. This could be especially beneficial to younger children, who are learning to make independent food decisions and can be helped and guided by concerned parents.

While NRCs show promise in positively influencing the dietary choices of children, it is worth noting some concerns. One concern is the possible psychological impact of observing and reporting students' eating behavior to parents. Recalling the BMI Report Cards of Arkansas, overweight students did report being embarrassed by measurement. Nutrition Report Cards, while less incendiary, could still make some students feel uncomfortable.

Another possible area of concern is strategic student response. If NRCs focus on healthy items taken, such as fruits and vegetables, students may take more healthy items but not actually eat them. Such a reaction by students would undermine the effectiveness of the report card and increase costs to school lunchrooms. While responding strategically to less healthy items on a report card would be more difficult, students could find other means for acquiring less healthy items, such as vending machines or school stores. Being aware of these possible strategic responses can inform research design for future implementation. Despite these challenges, the NRC holds promise for creating an effective and cost-efficient way to involve parents in their child's school nutrition behavior.

## Supporting Information

Appendix S1 **Sample of a Nutrition Report Card.**
(DOCX)

Appendix S2 Sample Code (STATA) to Program Food Service Computer to Send Weekly Nutrition Report Cards to Parents.
(DOCX)
