# Correction: Nutrition Report Cards: An Opportunity to Improve School Lunch Selection

**DOI:** 10.1371/annotation/d05663ff-3e52-4a37-bd13-829c01c80007

**Published:** 2013-11-15

**Authors:** Brian Wansink, David R. Just, Richard W. Patterson, Laura E. Smith

A funding organization and grant were incorrectly omitted from the Funding Statement. The Funding Statement should read: "This study was funded through USDA/ERS Grant #59-5000-0-0072. The funders had no role in study design, data collection and analysis, decision to publish, or preparation of the manuscript."

