# Supplementary material for: Nutrition Report Cards: An Opportunity to Improve School Lunch Selection
Source: PLoS One. 2013 Oct 2;8(10):e72008. doi: 10.1371/journal.pone.0072008 (PMC3788775; doi:10.1371/journal.pone.0072008)
Supplement: Appendix S1 — Sample of a Nutrition Report Card. (DOCX) [file pone.0072008.s001.docx]

**Appendix S1. Sample of a Nutrition Report Card**

Dear Parent/Guardian,

Thank you for participating in our study. Here is a summary of the lunch room purchases your child, First, Last, made during the week of May 29 - June 3. The weekly summary is followed by a day-by-day report:

Total Meals: **4**

Total White milk: **0**

Total Flavored (Chocolate or Strawberry): **4**

Bottled Water: **0**

Total Fruit/Vegetable Sides: **6**

Total Starchy Sides (Bread, Rice, Potatoes, or Beans): **0**

Ice Cream: **3**

Cookies: **1**

Chips: **0**

Other Snacks: **0**

| **Item Type** | **Monday** | **Tuesday** | **Wednesday** | **Thursday** | **Friday** | **Total** |
| --- | --- | --- | --- | --- | --- | --- |
| **Complete USDA Meal** | 1 | 1 | 1 | 0 | 1 | 4 |
| **White Milk** | 0 | 0 | 0 | 0 | 0 | 0 |
| **Flavored Milk** | 1 | 1 | 1 | 0 | 1 | 4 |
| **Fruit/Vegetables** | 2 | 0 | 2 | 0 | 2 | 6 |
| **Starchy Sides** | 0 | 0 | 0 | 0 | 0 | 0 |
| **Ice Cream** | 1 | 1 | 0 | 1 | 0 | 3 |
| **Cookies** | 0 | 0 | 1 | 0 | 0 | 1 |
| **Chips** | 0 | 0 | 0 | 0 | 0 | 0 |
| **Water** | 0 | 0 | 0 | 0 | 0 | 0 |
| **Other Snacks** | 0 | 0 | 0 | 0 | 0 | 0 |

You are receiving this E-mail because you enrolled your child in a study through ____________ University. Thank you for participating in our study.

Sincerely,
