# Supplementary material for: Nutrition Report Cards: An Opportunity to Improve School Lunch Selection
Source: PLoS One. 2013 Oct 2;8(10):e72008. doi: 10.1371/journal.pone.0072008 (PMC3788775; doi:10.1371/journal.pone.0072008)
Supplement: Appendix S2 — Sample Code (STATA) to Program Food Service Computer to Send Weekly Nutrition Report Cards to Parents. (DOCX) [file pone.0072008.s002.docx]

**Appendix S2. Sample Code (STATA) to Program Food Service Computer**

**to Send Weekly Nutrition Report Cards to Parents**

It is possible that school POS systems are able to provide data in a format that is report card-ready, but it is likely that reformatting will be required. To generate the NRC, the data file must contain one row entry per individual and one column for each variable. Item-level data, the most common type of data available, needs to be collapsed, reshaped, and merged with participant information. An example of how data can be reformatted appears below. This study used Stata 11 which can serve as a template for other computer languages. Once the data were reformatted, they were read into a report card shell formatted using a Microsoft Mail Merge Document file. Microsoft Outlook was then programed to e-mail the report cards to participating families. Once programmed, this took approximately 30 minutes, but the time could be further reduced with additional programming or with a template.

STATA Code

clear

*set directory

cd C:\Users\*******

****************************************

************ read in all files from directory

local i=0

cap erase joined06_03.dta

local files: dir . files "*.csv"

foreach f of local files {

drop _all

insheet using `f'

drop notes1

if `i'>0 append using joined06_03

save joined06_03, replace

local i=1

}

************ generate dummy variables for nutrition report card foods

gen chips=item=="Chips"

gen cookies=item=="Cookies"

gen ice_cream=item=="Ice Cream"

gen meal_i=item=="Meal"

gen white_milk=item=="Milk White"

gen other_snack=item=="Nutty bars"

gen water=item=="Water"

gen fruit=item=="lunch - Fruit/Veggie"

gen starch=item=="lunch - Starchy Side"

************ adjust for multiple items in line

global items "meal_i chips cookies ice_cream white_milk other_snack water fruit starch"

foreach x in $items {

replace `x'= `x'*itemcount if itemcount!=.

}

************ format date variable

gen date=date(transdate, "MDY")

format date %tdDay_Month_dd

drop if missing(date)

************ collapse data by day if creating reports with daily information

collapse (sum) $items, by(id date)

************ for convenience of interpretation we replace date with first day in week==1,

replace date=date-18777

reshape wide $items, i(id) j(date)

************ if no school on date i use following correction

foreach x in $items {

gen `x'i=0

}

************ flavored milk was a defualt for any meal so this loop accounts for all the meals that did not take white milk

forvalues i=1/5{

gen flavored_milk`i'=0

replace flavored_milk`i'=meal_i`i'-white_milk`i'

}

************ aggregate totals

foreach x in $items flavored_milk {

gen `x'_total=0

forvalues i=0/4 {

replace `x'`i'=0 if missing(`x'`i')

replace `x'_total=`x'_total+`x'`i' if `x'`i'!=.

}

}

************ merge in personal information, such as E-mail addresses

merge 1:1 id using profiles

************ output file in format that can be referenced in a mail merge

outsheet using C:\Users\Rich\Documents\fbl_research\Waverly\output06_03.csv, c replace
